# SH2D4A promotes centrosome maturation to support spindle microtubule formation and mitotic progression

**DOI:** 10.1038/s41598-023-29362-w

**Published:** 2023-02-04

**Authors:** Ryuzaburo Yuki, Yuki Ikeda, Ryuji Yasutake, Youhei Saito, Yuji Nakayama

**Affiliations:** Department of Biochemistry & Molecular Biology, Kyoto Pharmaceutical University, 5 Misasagi-Nakauchi-cho, Yamashina-ku, Kyoto, 607-8414 Japan

**Keywords:** Mitosis, Cell division, Mitotic spindle

## Abstract

Mitotic progression requires the precise formation of spindle microtubules based on mature centrosomes. During the G2/M transition, centrosome maturation progresses, and associated microtubules bundle to form mitotic spindle fibers and capture the chromosomes for alignment at the cell equator. Mitotic kinases-induced phosphorylation signaling is necessary for these processes. Here, we identified SH2 domain-containing protein 4A (SH2D4A/PPP1R38) as a new mitotic regulator. SH2D4A knockdown delays mitotic progression. The time-lapse imaging analysis showed that SH2D4A specifically contributes to the alignment of chromosomes. The cold treatment assay and microtubule regrowth assay indicated that SH2D4A promotes microtubule nucleation to support kinetochore–microtubule attachment. This may be due to the centrosome maturation by SH2D4A via centrosomal recruitment of pericentriolar material (PCM) such as cep192, γ-tubulin, and PLK1. SH2D4A was found to be a negative regulator of PP1 phosphatase. Consistently, treatment with a PP1 inhibitor rescues SH2D4A-knockdown-induced phenotypes, including the microtubule nucleation and centrosomal recruitment of active PLK1. These results suggest that SH2D4A is involved in PCM recruitment to centrosomes and centrosome maturation through attenuation of PP1 phosphatases, accelerating the spindle formation and supporting mitotic progression.

## Introduction

Mitotic cell division equally separates the replicated chromosomes and inherits them to daughter cells^1,2^. The centrosome is a fundamental membraneless organelle that supports cell division by forming bipolar spindles^3,4^. Centrosome maturation occurs during mitotic entry, with pericentriolar material (PCM) such as Cep192/SPD-2 and pericentrin accumulating around the centrioles^5,6^. The recruitment of γ-tubulin ring complex (γTuRC), one of the PCMs, is especially important for the nucleation of centrosomal microtubules. Spindle microtubules dynamically polymerize and depolymerize following centrosome maturation to capture the sister chromatid and transport the chromosomes to the spindle equator. Once all kinetochores have properly attached to the spindle microtubules, the spindle assembly checkpoint (SAC) is inactivated, and chromosomes are segregated^7^. Phosphorylation signaling governs whole mitotic processes^8^. Deregulation of the balance between phosphorylation and de-phosphorylation results in the collapse of mitotic processes^9^. At the mitotic entry, mitotic kinases are activated to orchestrate mitotic events, while the activity of Serine/Threonine (Ser/Thr) protein phosphatases such as protein phosphatase 1 (PP1) and protein phosphatase 2A (PP2A) is attenuated^10,11^. Cyclin-dependent kinase 1 (CDK1) directly phosphorylates the PP1 catalytic subunits α/β/γ at the C-terminal threonine residue and represses the phosphatase activities, causing the superiority of phosphorylation signaling by feed-forward loop in mitosis^12,13^.

Centrosome maturation is regulated by phospho-signaling. Polo-like kinase 1 (PLK1) and Aurora A kinase are PCM components and act as important maturation factors^14^. Centrosomal PLK1 promotes PCM expansion^15^, particularly γTuRC accumulation, via Cep192 phosphorylation and accelerates the nucleation of spindle microtubules^5,16,17^. PLK1 recruitment at centrosomes is mediated by its kinase activity^18^ and Aurora A activity^5^. PLK1 is activated by phosphorylation at Thr-210 in its activation loop by active Aurora A kinase^19^, while PP1 phosphatases de-phosphorylate PLK at this site and Aurora A to weaken those kinase activities^20–22^.

The PP1 activities are directly regulated by regulatory subunits^23,24^. SH2 domain-containing protein 4A (SH2D4A/PPP1R38) is a member of SH2 signaling protein family and is involved in the IL-6 signaling in cancer cells^25^. Moreover, SH2D4A binds to PP1α and inhibits its phosphatase activity^26^, suggesting that SH2D4A is one of the PP1 regulatory subunits. Considering the fact that the precise regulation of PP1 phosphatases is important for mitotic progression, SH2D4A may be involved in the mitotic processes; however, the role of SH2D4A in mitosis is completely unexplored.

In this study, we found that SH2D4A contributes to mitotic progression. The time-lapse imaging revealed that SH2D4A is important for prometaphase progression. SH2D4A is involved in the nucleation of spindle microtubules at centrosomes to promote construction of cold-stable microtubules. SH2D4A promotes centrosome maturation via PCM expansion, which includes Cep192, γ-tubulin, active PLK1, and active Aurora A. Furthermore, treatment with the PP1/PP2A inhibitor calyculin A recovered the decrease in the level of active PLK1 at centrosomes and microtubule nucleation caused by SH2D4A knockdown. Given that SH2D4A binds to PP1α/β in mitosis, SH2D4A participates in the mitotic progression through promoting centrosome-directed spindle assembly via inhibiting PP1 phosphatases.

## Results

### Role of SH2D4A in mitotic progression

**Figure 1:**
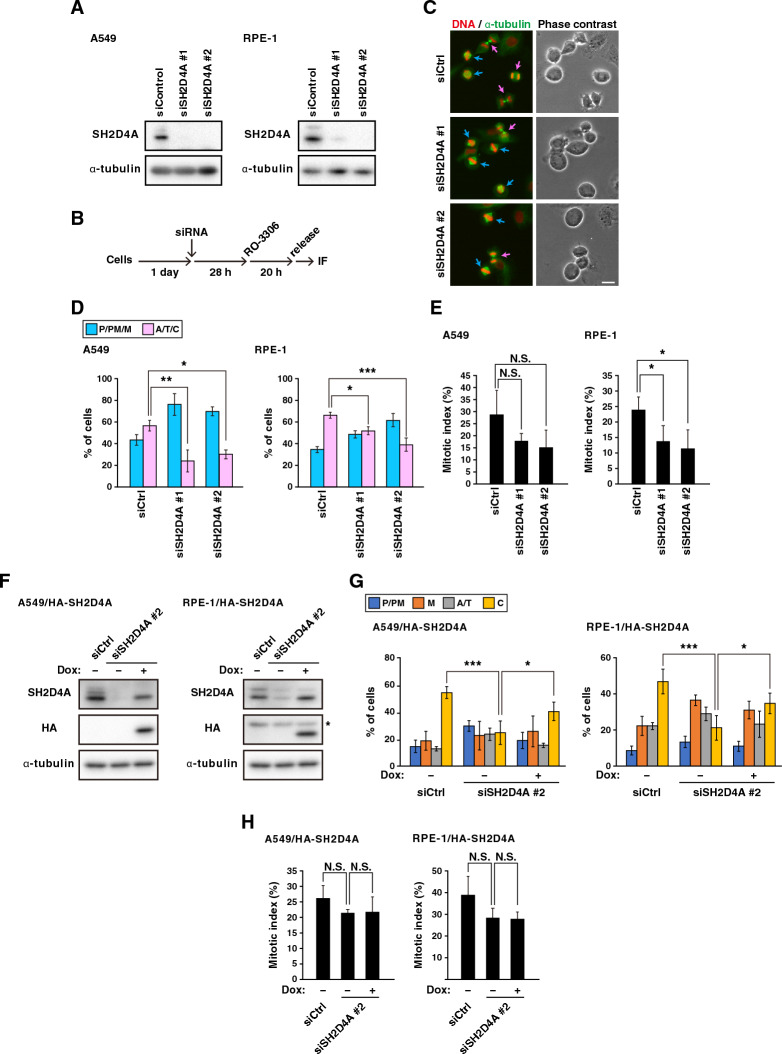
SH2D4A knockdown delays mitotic progression. (**A**) A549 or RPE-1 cells were transfected with control siRNA (siCtrl) or SH2D4A-targeting siRNAs (siSH2D4A #1 and #2). At 48 h after transfection, Western blot analysis was performed with indicated antibodies. (**B**–**E**) At 28 h after siRNA transfection, A549 or RPE-1 cells were treated with 6 or 8 µM RO-3306 for 20 h, washed with PBS(+), and cultured for 1 h or 45 min, respectively. Then, the cells were fixed and stained for α-tubulin (green) and DNA (red). (**B**) A schematic depiction of the synchronization method is shown. (**C**) Representative images of mitotic A549 cells are shown. The mitotic cells were classified into two groups (see “Methods”). Blue and pink arrows show the cells before and after anaphase onset, respectively. Scale bar, 20 µm. The percentages of cells of each group (**D**) or the mitotic indices (**E**) are plotted as the mean ± SD of more than three independent experiments (n > 168 in panel (**D**), n > 999 in panel (**E**)). (**F**) A549 or RPE-1 cells expressing inducible HA-SH2D4A (A549/HA-SH2D4A or RPE-1/HA-SH2D4A) were transfected with siCtrl or siSH2D4A #2 with or without 0.5 µg/mL (A549/HA-SH2D4A) or 0.3 µg/mL (RPE-1/HA-SH2D4A) Doxycycline (Dox). At 48 h after transfection, Western blot analysis was performed as in panel A. An asterisk indicates a non-specific band. (**G**, **H**) At 28 h after siRNA transfection, A549/HA-SH2D4A or RPE-1/HA-SH2D4A cells were treated with 6 or 8 µM RO-3306 for 20 h with or without Dox, washed, and cultured for 1 h or 50 min, respectively. The cells were stained for α-tubulin and DNA, and the mitotic cells were classified into four groups (see “Methods”) The percentages of cells of each group (**G**) or the mitotic indices (**H**) are plotted as the mean ± SD of four independent experiments (n > 214 in panel G, n > 1000 in panel (**H**)). Asterisks indicate significant differences (Dunnett’s test in panel (**D**) and (**E**), Tukey’s test in panel (**G**) and (**H**), * *p* < 0.05; ** *p* < 0.01; *** *p* < 0.001; N.S., not significant). Full blots are shown in Fig. S4.

Our previous report showed that mitotic progression can be monitored in a time-dependent manner after release from G2/M arrest that is induced by treatment with a reversible CDK1 inhibitor RO-3306^27^. To examine whether SH2D4A had a role in mitotic progression, we knocked down SH2D4A by two different sets of siRNAs in A549 cells and hTERT-immortalized retinal pigment epithelial RPE-1 cells (Fig. 1A) and examined mitotic progression using RO-3306 (Fig. 1B). We classified mitotic cells into two mitotic phases: before (prophase/prometaphase/metaphase) and after (anaphase/telophase/cytokinesis) chromosome segregation. In A549 cells (Fig. 1D, left), one-hour after release, about one half of control siRNA-treated mitotic cells (siCtrl) underwent chromosome segregation; however, more than two-thirds of SH2D4A-knockdown cells (siSH2D4A #1 and siSH2D4A #2) did not (Fig. 1C, D). Furthermore, similar delay in mitotic progression was observed in RPE-1 cells upon SH2D4A knockdown (Fig. 1D, right). One can suppose that this delay would be due to either a delay in mitotic progression or a delay in mitotic entry. To investigate this, we quantified prophase and prometaphase cells to see if knockdown cells entered mitosis later than the control cells. The percentage of prometaphase cells in A549 cells was comparable between the control and SH2D4A-knockdown cells 5 or 10 min after release from G2/M arrest (Fig. S1A), excluding the possibility that the increase in the cells before chromosome segregation (Fig. 1D) was due to a delay in the mitotic entry. Therefore, a decrease in mitotic index caused by SH2D4A knockdown is unlikely to be due to a delay in mitotic entry (Figs. 1E, S1B, C). To exclude the possibility of siRNA off-targets, we established the A549 or RPE-1 cell lines capable of inducible expression of HA-tagged SH2D4A by Doxycycline (Dox) treatment (A549/HA-SH2D4A or RPE-1/HA-SH2D4A cells). Endogenous SH2D4A was clearly knocked down by siSH2D4A #2, which targets the 3’-untranslated region (3’UTR) of the SH2D4A mRNA, whereas Dox addition induced expression of HA-tagged SH2D4A (Fig. 1F). We investigated mitotic progression using these cell lines by categorizing mitotic cells into four mitotic phases: prophase/prometaphase (P/PM), metaphase (M), anaphase/telophase (A/T), and cytokinesis (C). SH2D4A knockdown reduced the percentage of cells that progressed to cytokinesis, but Dox addition significantly rescued the decrease in both cell lines (Fig. 1G). The SH2D4A re-expression had no effect on mitotic indices (Fig. 1H). Collectively, these results suggest that SH2D4A is important for mitotic progression.

### Delay in the chromosome alignment by SH2D4A knockdown

**Figure 2:**
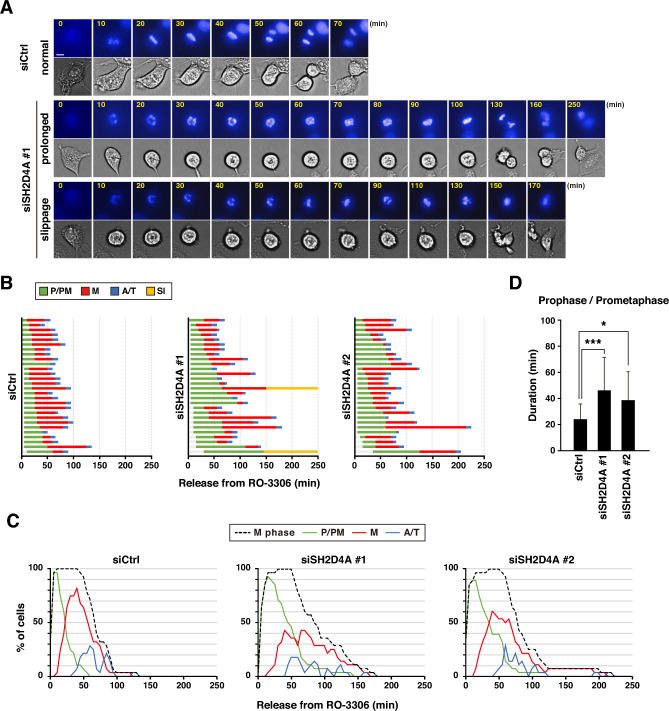
SH2D4A knockdown prolongs prometaphase duration. (**A**–**D**) A549 cells were transfected with control siRNA (siCtrl) or SH2D4A-targeting siRNAs (siSH2D4A #1 and #2). At 28 h after siRNA transfection, cells were treated with 6 µM RO-3306 for 20 h and washed with PBS(+). Then, the cells were monitored for 250 min by time-lapse imaging with 0.1 µM Hoechst 33342. (**A**) Images show typical phenotypes of siRNA-treated mitotic cells: cells that exhibit normal mitosis (normal mitosis), the prolonged-duration of prophase/prometaphase (prolonged), and the mitotic exit without chromosome segregation (slippage). Scale bar, 10 µm. (**B**) The duration of each mitotic phase is shown: prophase/prometaphase (P/PM: from nuclear envelope breakdown to chromosome condensation, green), metaphase (M: chromosome alignment, red), anaphase/telophase (A/T: from anaphase onset to cleavage furrow ingression, blue), and slippage (Sl: interphase cells that undergo mitotic slippage, yellow). In total, 28 mitotic cells were examined. (**C**) The percentages of mitotic cells (black), prophase and prometaphase (green), metaphase (red), and anaphase and telophase cells (blue) at the indicated times are plotted. (**D**) The duration time of prophase/prometaphase in each group is plotted as the mean ± SD from a representative experiment of two independent experiments. Asterisks indicate significant differences (Dunnett’s test, * *p* < 0.05, *** *p* < 0.001).

To examine which sub-phase SH2D4A regulated, we performed time-lapse imaging with staining of DNA by Hoechst 33342 after release from RO-3306 block. DNA staining could visualize the mitotic sub-phases (Fig. 2A): prophase/prometaphase cells (P/PM) having condensed chromosomes, metaphase cells (M) exhibiting chromosome alignment, and anaphase/telophase cells (A/T) having segregated sister chromatids. By monitoring the mitotic progression from the mitotic entry to the mitotic exit, the exact duration time of cell division and each mitotic sub-phase can be measured. During the time-lapse imaging analysis, mitotic entry was determined by the onset of an increase in the fluorescence intensity of Hoechst 33342, which indicates the beginning of chromosome condensation. Mitotic exit was determined by completion of furrow ingression. The time from mitotic entry to mitotic exit was 70.5 min in control cells, but its time was prolonged to 88.6 min and 87.5 min in siSH2D4A #1- and siSH2D4A #2-treated cells, respectively (Fig. 2B). Furthermore, the percentage of each sub-phase cells were plotted over time (Fig. 2C). The peak of prophase/prometaphase was almost unchanged regardless of SH2D4A knockdown, confirming that the timing of mitotic entry was not affected by SH2D4A knockdown, as previously described above. In contrast, the peak of metaphase cells was lowered and broadened by SH2D4A knockdown. Actually, SH2D4A knockdown prolonged the duration time of prophase/prometaphase (Fig. 2D).

In siSH2D4A #1-treated cells, 7% cells exhibited mitotic slippage, which incompletely ends mitosis without chromosome segregation (Fig. 2B), after delay in prophase/prometaphase and metaphase. Because mitotic slippage was not observed in siSH2D4A #2-treated cells, this effect might be an off-target effect upon treatment with siSH2D4A #1.

### Effect of SH2D4A knockdown on spindle microtubule formation

**Figure 3:**
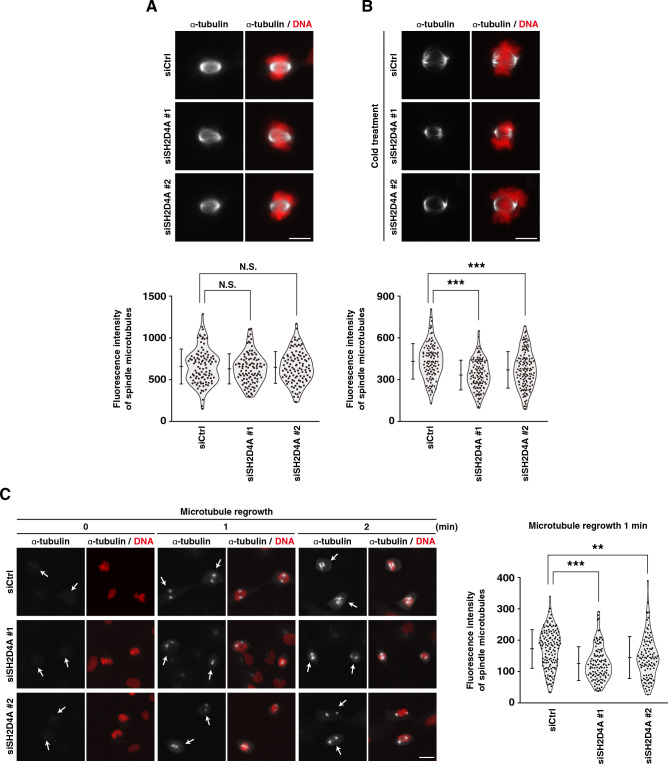
SH2D4A regulates the spindle formation at prometaphase. A549 cells were transfected with control siRNA (siCtrl) or SH2D4A-targeting siRNAs (siSH2D4A #1 and #2). At 28 h after siRNA transfection, cells were treated with 6 µM RO-3306 for 20 h. (**A**, **B**) RO-3306-treated cells were washed with PBS(+) and cultured in fresh media for 15 min. Before (**A**) or after (**B**) incubation on ice for 5 min, cells were fixed and stained for α-tubulin (gray) and DNA (red). (Upper panels) Representative images of prometaphase cells are shown. Scale bar, 10 µm. (Lower panels) The fluorescence intensity of α-tubulin was measured and plotted as the mean ± SD from a representative experiment of two independent experiments (n > 102). Violin plots with data points are also shown. (**C**) RO-3306-treated cells were washed with PBS(+), cultured in fresh media for 10 min, and incubated on ice for 4 h. Before or after incubation at 37 °C for 1 or 2 min, cells were fixed and stained for α-tubulin (gray) and DNA (red). (Left) Representative images are shown, and the white arrows indicate the prometaphase cells. Scale bar, 10 µm. (Right) In the cells incubated at 37 °C for 1 min, the fluorescence intensity of α-tubulin was measured and plotted as the mean ± SD from a representative experiment of two independent experiments (n > 106). Violin plots with data points are also shown. Asterisks indicate significant differences (Dunnett’s test, ** *p* < 0.01, *** *p* < 0.001; N.S., not significant).

In prometaphase, mature centrosome-derived mitotic microtubules continuously polymerize and depolymerize to bidirectionally attach to the kinetochore for bipolar spindle formation and align chromosomes at the cell equator. Since kinetochore-attached microtubules are cold stable compared with the other unattached microtubules, we first examined the effect of SH2D4A knockdown on the microtubule stability to scrutinize a role in microtubule dynamics. After release from RO-3306 block, the cells were proceeded to the prometaphase, and then subjected to cold treatment for 5 min. Without cold treatment, SH2D4A knockdown had almost no effect on the fluorescence intensity of α-tubulin (Fig. 3A). However, cold treatment decreased the fluorescence intensity, and SH2D4A knockdown further decreased it (Fig. 3A, B). This suggests that SH2D4A is involved in the microtubule stability via adequate kinetochore attachment of spindle microtubule. Next, we performed microtubule regrowth assay to examine the role of SH2D4A in microtubule polymerization. Prometaphase cells synchronized by RO-3306 treatment were subjected to cold treatment for 4 h for complete depolymerization of microtubules (Fig. 3C, left, 0 min) and then cultured with pre-warmed medium for 1–2 min to trigger the microtubule polymerization. One- or two-minutes incubation allows polymerization of microtubule, and cells show a radial array of microtubules (Fig. 3C, left, 1 and 2 min). In SH2D4A-knockdown cells, the fluorescence intensity of α-tubulin was decreased in the cells incubated at 37 °C for 1 min (Fig. 3C), suggesting that SH2D4A promotes microtubule polymerization. Collectively, these results suggest that SH2D4A promotes microtubule polymerization to support the spindle microtubule formation and kinetochore–microtubule attachment in prometaphase.

### Involvement of SH2D4A in centrosome maturation

**Figure 4:**
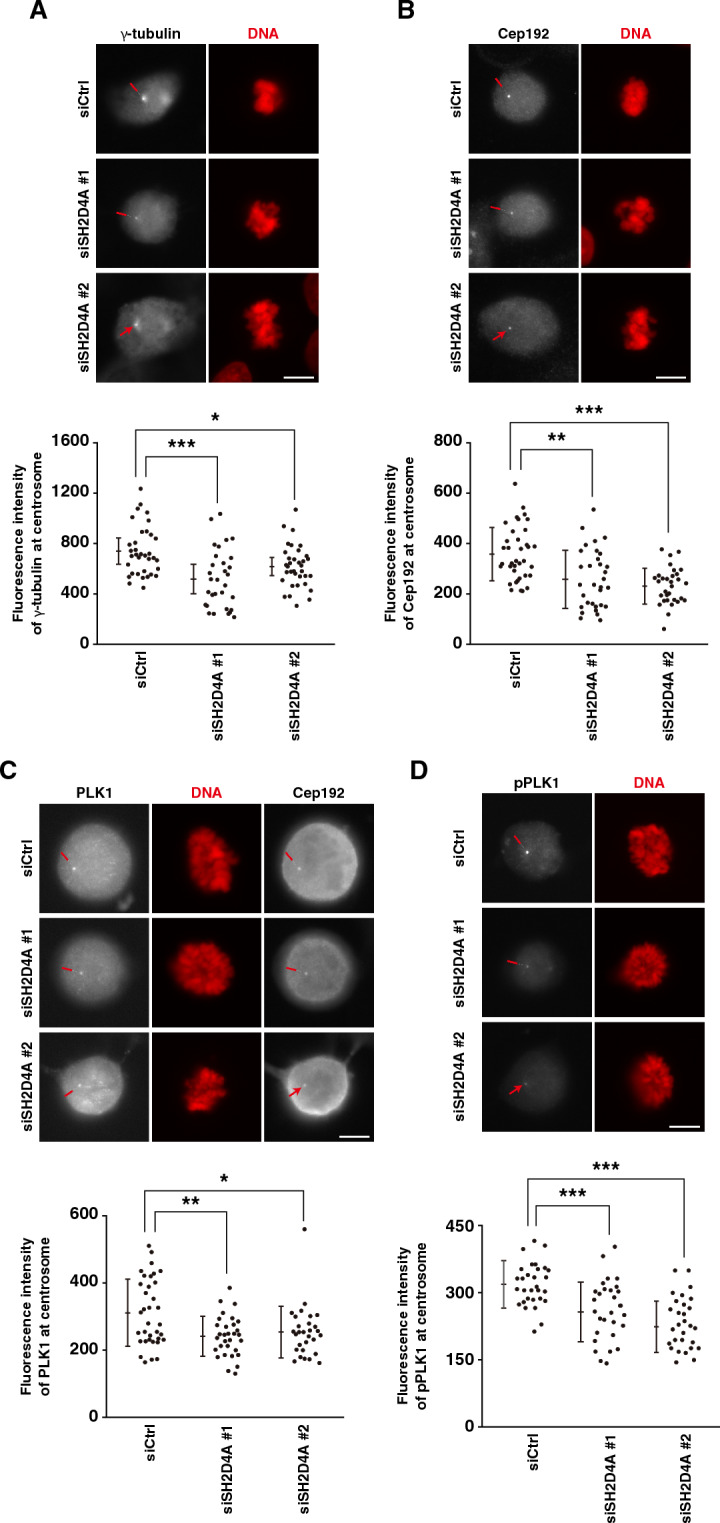

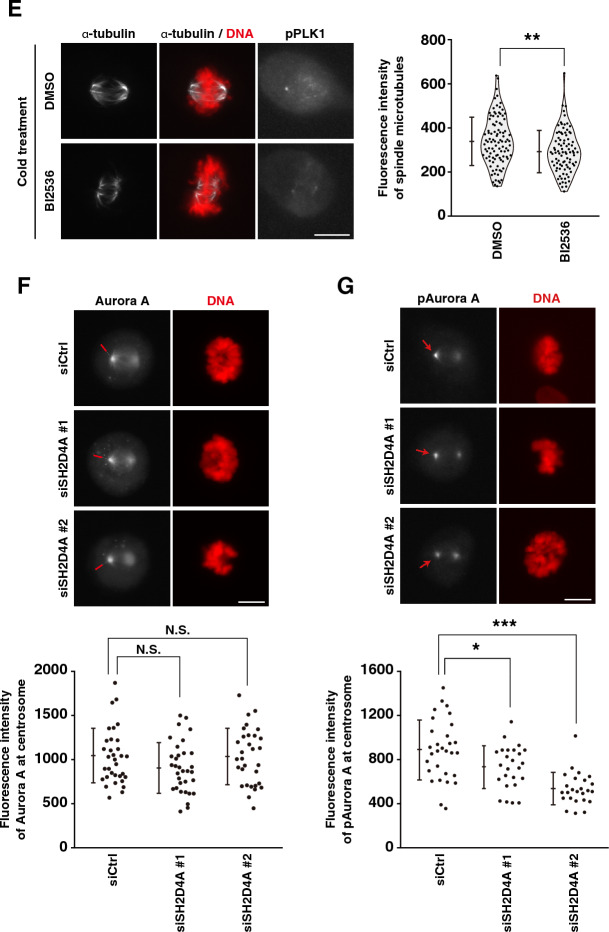
SH2D4A promotes the centrosome maturation. (**A**–**D**, **F**, **G**) A549 cells were transfected with control siCtrl, siSH2D4A #1, or siSH2D4A #2. At 28 h after siRNA transfection, cells were treated with 6 µM RO-3306 for 20 h. RO-3306-treated cells were washed with PBS(+) and cultured in fresh media for 30 min. Then, the cells were fixed and stained for indicated antibodies (gray) and DNA (red). (Upper panels) Representative images of prometaphase cells are shown. The focus was set to one of the centrosomes, and the red arrows indicate the centrosome. Scale bar, 10 µm. (Lower panels) The fluorescence intensity of γ-tubulin (**A**), Cep192 (**B**), PLK1 (**C**), phospho-PLK1 (pT210) (**D**), Aurora A (**F**), or phospho-Aurora A (pT288) (**G**) at centrosomes in prometaphase per cell was measured (see “Methods”) and the average between the two centrosomes was plotted as the mean ± SD from a representative experiment of two independent experiments (n > 24). (**C**) The fluorescence signal of PLK1 was detected on both centrosomes and kinetochores, therefore Cep192 staining was used to detect centrosomal PLK1. (**E**) A549 cells were treated with 6 µM RO-3306 for 20 h with or without 100 nM BI2536 during the last 15 min. RO-3306-treated cells were washed with PBS(+), cultured in fresh media for 15 min with or without BI2536, and incubated on ice for 5 min. Then, the cells were fixed and stained for α-tubulin (gray), DNA (red), and phospho-PLK1 (pT210) (gray). (Left) Representative images of prometaphase cells are shown. Scale bar, 10 µm. (Right) The fluorescence intensity of α-tubulin was measured and plotted as the mean ± SD from a representative experiment of two independent experiments (n > 89). Violin plots with data points are also shown. Asterisks indicate significant differences (Dunnett’s test in panel (**A**), (**D**), and (**F**), Games–Howell test in panel (**B**), (**C**), and (**G**), Student's *t*-test in panel (**E**), * *p* < 0.05; ** *p* < 0.01; *** *p* < 0.001; N.S., not significant).

Nucleation of mitotic spindles at centrosomes requires a crucial step known as centrosome maturation that includes the centrosome growth and accumulation of PCM such as Cep192, γTuRC, and mitotic kinases including PLK1 and Aurora A^28^. Since SH2D4A promotes the nucleation of mitotic spindles at centrosomes (Fig. 3), we examined whether SH2D4A is involved in centrosome maturation. Cep192 is known to recruit PLK1 and Aurora A to centrosomes, where these kinases are then activated. Activated PLK1 phosphorylates Cep192 to associate with γTuRC, leading to PCM expansion and microtubule nucleation^29^. We knocked down SH2D4A and quantified the fluorescence intensity of PCM proteins at centrosomes. SH2D4A knockdown partially decreased the intensity of γ-tubulin, a component of γTuRC, and Cep192 at centrosomes (Fig. 4A, B). Moreover, the fluorescence intensity of total PLK1 and active PLK1, which is phosphorylated form at Thr-210 in its activation loop by Aurora A kinase, was also decreased by SH2D4A knockdown (Fig. 4C, D), whereas the protein level of PLK1 did not change in whole cell lysates by SH2D4A knockdown (Fig. S2A). As PLK1 is required for microtubule nucleation by promoting γTuRC binding to Cep192, treatment with the PLK1 inhibitor BI2536 decreased the fluorescence intensity of α-tubulin in cold-treated prometaphase cells as well as SH2D4A knockdown (Fig. 4E). We also examined the recruitment of total Aurora A and its activated form, phospho-Aurora A at Thr-288, to centrosomes. SH2D4A knockdown reduced the fluorescence intensity of phospho-Aurora A but had no effect on total Aurora A (Fig. 4F, G). These results suggest that SH2D4A is involved in centrosome maturation to support spindle microtubule nucleation and thereby spindle microtubule formation. We hypothesized that SH2D4A would localize at centrosomes. However, overexpressed HA-tagged SH2D4A distributed throughout the cytoplasm, excluding the chromosome region, and did not show specific localization at centrosomes (Fig. S2B, C).

### SH2D4A-mediated recruitment of active PLK1 and microtubule nucleation via PP1 phosphatases in mitosis

**Figure 5:**
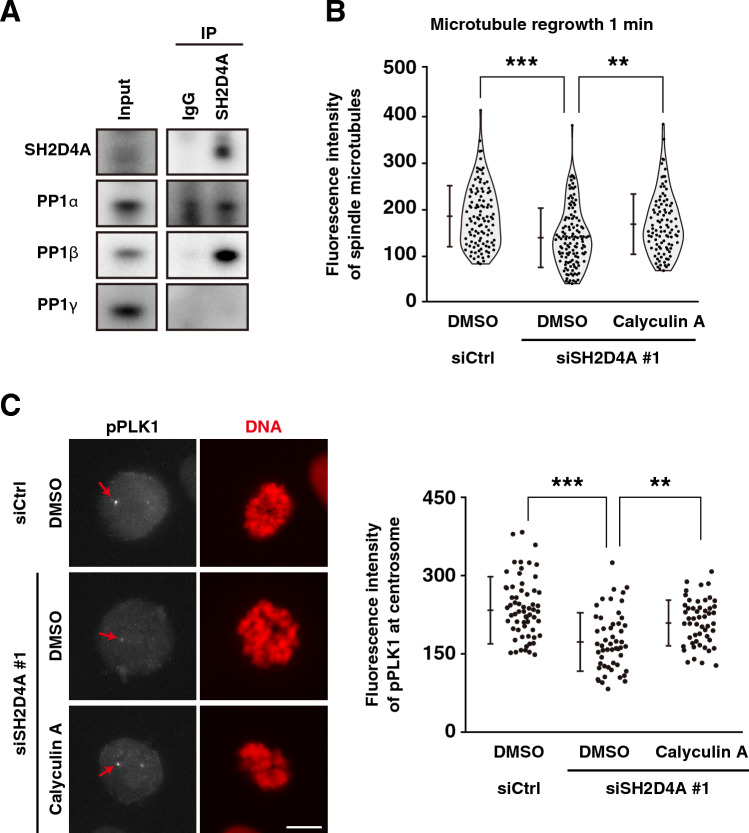
SH2D4A-mediated regulation of active PLK1 accumulation at centrosomes and microtubule nucleation through binding to PP1. (**A**) A549 cells were treated with 5 µM STLC for 16 h to arrest at mitosis. SH2D4A immunoprecipitates were subjected to Western blot analysis using indicated antibodies. (**B**, **C**) A549 cells were transfected with siCtrl or siSH2D4A #1. (**B**) At 28 h after siRNA transfection, cells were treated with 6 µM RO-3306 for 20 h, washed, cultured in fresh media for 10 min, and incubated on ice for 4 h with or without 2 nM calyculin A during the last 30 min. After incubation at 37 °C for 1 min with or without calyculin A, the cells were fixed and stained for α-tubulin and DNA. The fluorescence intensity of α-tubulin was measured and plotted as the mean ± SD from a representative experiment of two independent experiments (n > 100). Violin plots with data points are also shown. (**C**) At 28 h after siRNA transfection, cells were treated with 6 µM RO-3306 for 20 h with or without 2 nM calyculin A during the last 30 min. RO-3306-treated cells were washed, cultured in fresh media for 30 min with or without calyculin A. Then, the cells were fixed and stained for phospho-PLK1 (gray) and DNA (red). (Left) Representative images of prometaphase cells are shown. The focus was set to one of the centrosomes, and the red arrows indicate the centrosome. Scale bar, 10 µm. (Right) The fluorescence intensity of phospho-PLK1 at centrosomes in prometaphase per cell was measured and the average between the two centrosomes was plotted as the mean ± SD from a representative experiment of two independent experiments (n > 52). Asterisks indicate significant differences. Asterisks indicate significant differences (Tukey’s test, ** *p* < 0.01; *** *p* < 0.001). Full blots are shown in Fig. S4.

Considering that SH2D4A inhibits PP1 activity^26^ and PP1 inactivates PLK1 and Aurora A by de-phosphorylation^22^, PP1 phosphatases may be involved in the SH2D4A-mediated regulation of microtubule nucleation. Immunoprecipitation experiment showed that endogenous SH2D4A co-immunoprecipitated PP1α and PP1β catalytic subunits but not PP1γ in mitosis (Fig. 5A). Furthermore, treatment of cells with calyculin A, a PP1/PP2A inhibitor, recovered the attenuation of the microtubule nucleation caused by SH2D4A knockdown (Figs. 5B, S3). Furthermore, SH2D4A knockdown-induced decrease in the level of active PLK1 at centrosomes was also recovered by calyculin A treatment (Fig. 5C). These results suggest that SH2D4A-mediated recruitment of active PLK1 at centrosomes and subsequent microtubule nucleation involves PP1 phosphatases.

## Discussion

In this study, we explored a role of the PP1 regulatory subunit SH2D4A in mitosis. SH2D4A knockdown delayed mitotic progression especially in prometaphase, indicating that SH2D4A has a role in chromosome alignment. Because proper chromosome alignment necessitates adequate microtubule dynamics, we next investigated the effects of SH2D4A on mitotic spindle microtubules. Cold treatment and subsequent microtubule regrowth assay revealed that SH2D4A promotes kinetochore attachment of microtubules possibly through microtubule nucleation and/or polymerization. Centrosome maturation is a key step for ensuring proper mitotic spindle formation through microtubule nucleation. In agreement with this, SH2D4A knockdown suppressed the centrosome maturation, evaluated by the recruitment of γ-tubulin, cep192, PLK1, and active Aurora A. Finally, we investigated the effect on PP1, a protein negatively regulated by SH2D4A, and found that SH2D4A was co-precipitated with PP1α and β. PP1 inhibition reversed the SH2D4A knockdown-caused retardation of microtubule nucleation and the decrease in PLK1 phosphorylation level. Taken together, we conclude that SH2D4A represses PP1 phosphatase activity and thereby contributes to centrosome maturation, supporting formation of the mitotic spindle microtubules.

Mitotic progression is slowed down by several defects, including the delay in chromosome alignment, persistent SAC activation, and cleavage furrow disorganization^30^. SH2D4A knockdown delays in mitotic progression via retardation of the prometaphase events (Figs. 1, 2). No prolongation was observed in the other sub-phases (Fig. 2), suggesting that persistent SAC activation and cleavage furrow disorganization did not occur in SH2D4A-knockdown cells. Mitotic prolongation caused by SH2D4A knockdown is attributed to chromosome alignment delays because SH2D4A knockdown delays centrosome maturation and microtubule nucleation.

SH2D4A interacts with PP1α and β catalytic subunits, according to our findings (Fig. 5A). It is unknown which catalytic subunit is involved in the SH2D4A-mediated regulation of centrosome maturation and mitotic progression. Previous research found that myosin phosphatase targeting subunit 1 (MYPT1) activates PP1β and reduces PLK1 phosphorylation at Thr-210, contributing to the repression of the PLK1 hyperactivation^20,21^. Partial knockdown of PLK1 leads to decrease in the γ-tubulin fluorescence intensity, whereas further MYPT1 knockdown restores it, indicating that PP1β-MYPT1 complex is involved in centrosome maturation via PLK1 regulation^21^. Thus, it is possible that SH2D4A-mediated PP1β inactivation would contribute to the mitotic progression and the precise balance of PP1 regulation by MYPT1 and SH2D4A might be important for centrosome maturation. However, we cannot rule out the possibility that PP1α plays a role in the mitotic function of SH2D4A, because PP1α is thought to inhibit NIMA-related kinase 2 (Nek2) activity at centrosomes^31,32^, and Nek2 is required for centrosome separation and maturation^32,33^.

Centrosome maturation progresses during the G2/M transition to form a proper bipolar spindle^34^. On the basis of centriole-associated pericentrin, Cep192 gradually accumulates at centrosomes^5,35^. Aurora A then binds to Cep192, recruits PLK1 kinase to Cep192, and activates PLK1 by phosphorylating Thr-210. Activated PLK1 phosphorylates Cep192 and pericentrin to facilitate the expansion of PCM, especially γTuRC, leading to nucleation of microtubules. SH2D4A partially participates in these maturation processes (Fig. 4). We found that SH2D4A knockdown affected the Aurora A activity (Fig. 4F, G). Aurora A activity is controlled by auto-phosphorylation at Thr-288, which can be dephosphorylated by PP1 phosphatase^22^. Previous report showed that the PP1 regulatory subunit PNUTS inhibits PP1 activity to sustain the Aurora A phosphorylation and activity^36^. As PP1 phosphatases are involved in SH2D4A-mediated centrosome maturation (Fig. 5), SH2D4A may reduce PP1 phosphatase activity, thereby sustaining Aurora A activity and facilitating PCM expansion. To prove that the SH2D4A-mediated regulation of centrosome maturation involves PP1α or β, it is necessary to confirm whether PP1α or β is localized at centrosome and regulates Aurora A activity.

Spindle microtubules emerge from mature centrosomes and dynamically reform to capture kinetochores during prometaphase for chromosome alignment at the equatorial plane^37^. The fluorescence intensity of α-tubulin was reduced in cold-treated cells at 15 min after RO-3306 release (Fig. 3B), suggesting that SH2D4A knockdown weakens the kinetochore–microtubule attachment, resulting in spindle instability. Because the time point of analysis was early stage of prometaphase and SH2D4A knockdown slowed down the centrosome-directed microtubule nucleation (Fig. 3C), the stable kinetochore–microtubule attachment was not completed at 15 min after the RO-3306 release. Therefore, reduced microtubule stability was observed by SH2D4A knockdown at this time point. However, given that SH2D4A regulates PLK1 activity and that kinetochore-attached PLK1 stabilizes the kinetochore–microtubule attachment^38^, it is possible that SH2D4A knockdown-mediated PLK1 inactivation via PP1 hyperactivation would weaken the kinetochore–microtubule attachment.

Using the RO-3306 synchronization method, we investigated the role of SH2D4A in mitotic progression. After release from G2/M arrest, approximately 20–40% of control cells were observed to enter mitosis (Fig. 1E). Upon SH2D4A knockdown, the mitotic index tended to decrease, as calculated at early or latter time point after release (Figs. 1E, S1B, C), although this depends on the cell type. However, SH2D4A knockdown had no effects on the percentage of cells that progressed to prometaphase at 5 or 10 min after release from RO-3306 synchronization (Fig. S1A), and time-lapse imaging analysis showed that the peak of prophase/prometaphase was comparable between the control and SH2D4A-knockdown cells (Fig. 2C); therefore, it is thought that mitotic entry is unaffected in most cells that could enter mitosis. Given that SH2D4A regulates PLK1 and Aurora A activities (Fig. 4), both of which are required for mitotic entry^39–41^, it is likely that mitotic entry was impaired in a subpopulation of SH2D4A-knockdown cells, resulting in a decrease in the mitotic index.

We conclude that SH2D4A-mediated spindle formation involves regulation of PP1 phosphatases, because calyculin A rescued a decrease in microtubule nucleation caused by SH2D4A knockdown (Fig. 5). However, another downstream pathway of SH2D4A might be also involved in this microtubule regulation. SH2D4A is known to interact with the STAT3 transcription factor to inhibit dimerization and retain the cytoplasmic localization of STAT3, resulting in inhibition of IL-6 signaling^25^. It is of note that cytoplasmic STAT3 regulates microtubule dynamics in interphase in a manner independent of its dimerization and transcriptional activity. Inhibition of Stathmin, which destabilizes the microtubule minus end, by direct interaction of STAT3 may be involved in the promotion of the microtubule polymerization^42,43^. Furthermore, Stathmin overexpression or knockdown inactivates centrosomal PLK1^44^, suggesting that deregulation of Stathmin levels may negatively affect PLK1 function. Therefore, it is possible that SH2D4A may contribute to centrosome maturation and the formation of cold-stable microtubules in mitosis by, in part, modulating the interaction between STAT3 and Stathmin and thereby affecting PLK1 function.

*SH2D4A* gene is located on the short arm of chromosome 8 (8p.21–22). Recent research showed that, 8p.21–22 deletion is observed in more than 5% of primary colorectal cancers (CRCs), and the expression levels of 11 genes including SH2D4A are down-regulated^45^. The low expression levels of SH2D4A and 11 genes correlated with poor prognosis of CRCs. Furthermore, some cases of hepatocellular carcinomas (HCCs) with loss of chromosome 8p exhibits poor prognosis, in which six genes including SH2D4A are down-regulated^46,47^. SH2D4A inhibits HCCs proliferation through weakening the STAT signaling^25^, suggesting a possibility of SH2D4A as a tumor suppressor. Intriguingly, 8p.21–22-deleted CRCs belong to the micro satellite stable (MSS) type, and MSS type CRCs typically show chromosome instability (CIN) during the development of carcinogenesis^48^. Considering that mitotic aberration is one of the causes of CIN development^49^, it is speculated that loss of the SH2D4A-mediated mitotic regulation might be involved in those CIN development.

In conclusion, we demonstrate that SH2D4A promotes centrosome maturation and supports the spindle nucleation through PP1 phosphatases and contributes to adequate mitotic progression. Aurora A and PLK1 activities are regulated by PP1–regulatory subunit complex. However, it is obscure that SH2D4A and other regulatory subunits would cooperatively regulate these kinase activities. Since the PhosphoSitePlus proteomics shows that several serine and threonine residues of SH2D4A could be phosphorylated, it is possible that phosphorylation of SH2D4A modifies the activity against PP1 phosphatase. Further studies are required to reveal the precise spatiotemporal regulation of SH2D4A in mitotic progression.

## Methods

### Cells

A549 and Lenti-X 293 T (Clontech Laboratories, Mountain View, CA, USA) cells were cultured in Dulbecco’s modified Eagle’s medium (DMEM) containing 5% fetal bovine serum (FBS) with 20 mM HEPES–NaOH (pH 7.4) at 37 °C in 5% CO_2_. hTERT RPE-1 (CRL-4000; American Type Culture Collection, Manassas, VA, USA) cells were cultured in DMEM/Ham's F-12 (0846095; Nacalai Tesque, Kyoto, Japan) containing 10% FBS at 37 °C under 5% CO_2_.

To establish A549 cell lines capable of inducible overexpression of HA-tagged SH2D4A (A549/HA-SH2D4A cells), Lenti-X 293 T cells were co-transfected in a 35-mm dish with 1.2 µg of pLIX_402 vector harboring the HA-tagged SH2D4A cDNA, 0.8 µg of pCAG-HIVgp, and 0.8 µg of pCMV-VSV-G-RSV-Rev by using PEIMAX (Polysciences, Warrington, PA, USA). The medium was changed the next day after transfection. The virus-containing medium was harvested 48 h after medium change and passed through a 0.45-mm filter. A549 cells were infected with 80 µg/ml polybrene (MilliporeSigma, Burlington, MA, USA) and selected in 1 µg/mL puromycin (StressMarq Biosciences, Victoria, BC, Canada). After 1 week selection, the selected cells were re-infected and re-selected in puromycin. To establish RPE-1 cell lines capable of inducible overexpression of HA-tagged SH2D4A (RPE-1/HA-SH2D4A cells), Lenti-X 293 T cells were transfected as described above using Lipofectamine 2000 reagent (Invitrogen, Carlsbad, CA, USA Tris-buffered saline. At 16 h after transfection, 10 µM Forskolin (Fujifilm Wako Pure Chemicals, Osaka, Japan) was added to the medium. After 8 h, the virus-containing medium was harvested and passed through a filter. RPE-1 cells were infected with polybrene, and the infected cells were selected in 2 µg/ml puromycin.

### Plasmids

Human SH2D4A cDNA inserted into pcDNA3.1 + N-HA vector was purchased from Genscript (Piscataway, NJ, USA). HA epitope-tagged SH2D4A was subcloned in pENTR4-no-ccDB (686-1)[a gift from Eric Campeau & Paul Kaufman (Addgene plasmid # 17424; http://n2t.net/addgene:17424; RRID:Addgene_17424)^50^], and recombined with pLIX_402 (a gift from David Root, addgene plasmid #41394) lentiviral plasmids using the Gateway LR reaction according to the manufacture’s instruction (Invitrogen). The lentiviral packaging plasmids pCAG-HIVgp and pCMV-VSV-G-RSV-Rev were a gift from Dr Hiroyuki Miyoshi [Rikagaku Kenkyusho Foundation (RIKEN) BioResource Center, Ibaraki, Japan].

### siRNA

Ten picomoles of small interfering RNA (siRNA) per well of a 24-well plate were transfected to A549 and hTERT RPE-1 cells with using Lipofectamine 2000 reagent. siSH2D4A #1 (Hs02_00355436) and siSH2D4A #2 (Hs01_00096823) were purchased from MilliporeSigma.

### Antibodies

The primary antibodies were used for immunofluorescence (IF) and immunoblotting (IB) as follows: rat monoclonal α-tubulin (IF, 1:800; IB, 1:4000; MCA78G, Bio-Rad, Hercules, CA, USA), mouse monoclonal SH2D4A (IB, 1:500; sc-514170, Santa Cruz Biotechnology, Dallas, TX, USA), mouse monoclonal HA-tag (IB, 1:1000; IF, 1:2000; M180-3, Medical and Biological Laboratories, Tokyo, Japan), mouse monoclonal γ-tubulin (IF, 1:600; GTU-88, MilliporeSigma), rabbit polyclonal Cep192 (IF, 1:400; 18832-1-AP, Proteintech, Rosemont, IL, USA), mouse monoclonal PLK1 (IF, 1:200; ab17057, abcam, Cambridge, UK), mouse monoclonal PLK1 (IB, 1:1000; sc-17783, Santa Cruz Biotechnology), rabbit polyclonal phospho-PLK1 (pT210) (IF, 1:250; 618601, BioLegend, San Diego, CA, USA), mouse monoclonal Aurora A (IF, 400; 610938, BD Biosciences, Franklin Lakes, NJ, USA), rabbit monoclonal phospho-Aurora A (pT288) (IF, 1:200; #3079, Cell Signaling Technology, Danvers, MA, USA), mouse monoclonal PP1α (IB, 1:200; sc-271762, Santa Cruz Biotechnology), mouse monoclonal PP1β (IB, 1:500; sc-365678, Santa Cruz Biotechnology), mouse monoclonal PP1γ (IB, 1:500; sc-515943, Santa Cruz Biotechnology), rabbit polyclonal phospho-Ser/Thr (IB, 1:1000; PP2551, ECM biosciences, Versailles, KY, USA) antibodies. For IF, Alexa Fluor 488-, 555-, 647-labeled donkey anti-mouse, anti-rabbit, and anti-rat (1:800; Life Technologies, Carlsbad, CA, USA) IgG antibodies were used. For IB, horseradish peroxidase-conjugated anti-mouse (1:8000; 715-035-151, Jackson ImmunoResearch, West Grove, PA, USA), anti-rabbit (1:8000; 711-035-152, Jackson ImmunoResearch), and anti-rat (1:8000; 712-035-153, Jackson ImmunoResearch) IgG antibodies were used.

### Cell-cycle synchronization

To analyze the effects of knockdown or knockdown-rescue of SH2D4A on mitotic progression, cells were arrested at the G2/M border by treating cells with the CDK1 inhibitor RO-3306 for 20 h. The used concentration was 6 µM in A549 and A549/HA-SH2D4A cells, or 8 µM in RPE-1 and RPE-1/HA-SH2D4A cells. To release cells from the G2/M arrest, the cells were washed with prewarmed PBS supplemented with Ca^2+^ and Mg^2+^ [PBS(+)] three times on a water bath at 37 °C and incubated in a prewarmed medium. Subsequent incubation time was 60 min in A549 and A549/HA-SH2D4A cells, 45 min in RPE-1 cells, or 50 min in RPE-1/HA-SH2D4A cells. Then, the cells were fixed with 4% formaldehyde in PBS for 20 min at room temperature. The fixed cells were stained for α-tubulin and DNA and classified into some categories, based on the microtubule and chromosome morphologies. In Fig. 1D, prophase/prometaphase/metaphase (P/PM/M) and anaphase/telophase/cytokinesis (A/T/C). In Fig. 1G, prophase/prometaphase (P/PM), metaphase (M), anaphase/telophase (A/T), and cytokinesis (C). In Fig. S1, prophase (P), prometaphase (PM), and metaphase (M). The percentage of each category was calculated. Additionally, to examine the percentage of synchronized cells that could enter mitosis, the mitotic indices, which indicate the ratio of the number of mitotic cells to the total number of cells, are calculated in each experiment.

### Immunofluorescence microscopy

For α-tubulin staining, formaldehyde-fixed cells were permeabilized and blocked with PBS(−) containing 0.1% saponin and 3% bovine serum albumin (BSA) for 30 min, incubated with the primary antibody for 1 h, and subsequently with the secondary antibody for 1 h along with 1 µM Hoechst 33342 for DNA staining. For staining of overexpressed-SH2D4A, cells were fixed with 4% formaldehyde in PIPES, Triton, EGTA and MgCl_2_ (PTEM) buffer [2 mM PIPES (pH 6.8), 0.2% Triton X-100, 10 mM EGTA, 1 mM MgCl_2_] for 20 min at 37 °C, and stained as indicated above. For Aurora A, phospho-PLK1, or phosphor-Aurora A staining and quantification, cells were fixed with 100% MeOH for 5 min at − 30 °C, and stained as indicated above. For PLK1 staining and quantification, cells were fixed with 4% formaldehyde in PTEM buffer for 20 min at 37 °C, and stained with Cep192 to distinguish between centrosomal and kinetochore PLK1, as indicated above. For Cep192 and γ-tubulin staining and quantification, cells were fixed with 100% MeOH for 5 min at − 30 °C, permeabilized with PBS(−) containing 0.2% Triton X-100 for 10 min at room temperature, blocked with PBS(−) containing 0.1% Triton X-100 and 3% BSA for 30 min, and incubated with the primary antibody and secondary as indicated above. The fluorescence images were obtained using an IX-83 fluorescence microscope (Olympus, Tokyo, Japan) equipped with a × 40/0.75 NA or a × 60/1.42 NA oil-immersion objective lens (Olympus). The optical system included a U-FUNA filter cube (360–370 nm excitation, 420–460 nm emission), a U-FBNA cube (470–495 nm excitation, 510–550 nm emission), a U-FRFP cube (535–555 nm excitation, 570–625 nm emission), and a U-DM3-CY5 cube (600–650 nm excitation, 670–740 nm emission) to observe Hoechst 33342, Alexa Fluor 488, Alexa Fluor 555, and Alexa Fluor 647 fluorescence, respectively. The mean fluorescence intensity of centrosomal proteins was calculated as the difference between a circular area of interest and an identically sized neighboring area^5^, using the ImageJ software (NIH, Bethesda, MD, USA). The captured images were edited using Photoshop CC and Illustrator CC software (Adobe, San Jose, CA, USA).

### Cold treatment

Staining of cold-stable microtubule was performed as described previously^51^. Briefly, the cells were released from the RO-3306-mediated G2/M arrest and incubated in a prewarmed medium for 15 min to progress into prometaphase. Then, the cells were incubated on ice for 5 min to depolymerize microtubules. Cold-treated cells were fixed with prewarmed 4% formaldehyde in PTEM buffer for 20 min in a water bath at 37 °C and stained for α-tubulin and DNA. The mean fluorescence intensity of α-tubulin staining was calculated as the difference between a circular area containing whole spindle microtubules and an identically sized background area using the ImageJ software.

### Microtubule regrowth

Analysis of the microtubule regrowth was performed as described previously^51^. Briefly, the cells were released from the RO-3306-mediated G2/M arrest and incubated in a prewarmed medium for 10 min to progress into prometaphase. Then, the cells were incubated on ice for 4 h to completely depolymerize microtubules, which was confirmed by staining for α-tubulin. Immediately after incubation of the cold-treated cells with a prewarmed medium for 1–2 min to polymerize the microtubule, the cells were fixed with a prewarmed 4% formaldehyde in PTEM buffer for 20 min in a water bath at 37 °C and stained for α-tubulin and DNA. The mean fluorescence intensity of α-tubulin staining was calculated as the difference between a circular area containing whole spindle microtubules and an identically sized background area using the ImageJ software.

### Time-lapse imaging

Time-lapse imaging was performed as described previously^52^. Briefly, synchronized A549 cells at G2/M border by RO-3306 treatment were washed with prewarmed PBS(+) three times in a water bath at 37 °C and incubated in a prewarmed medium with 0.1 µM Hoechst 33342. Then, time-lapse imaging was performed using a high-content imaging system (Operetta, PerkinElmer Life Sciences, Waltham, MA, USA) at 37 °C in 5% CO_2_.

### Western blotting

Western blotting was performed as described previously^53,54^. Briefly, the cells were lysed in sodium dodecyl sulfate (SDS) sample buffer containing protease inhibitors [10 μg/mL aprotinin (Fujifilm Wako Pure Chemicals), 4 μg/mL pepstatin A (Peptide Institute, Inc, Osaka, Japan), 10 μg/mL leupeptin (Nacalai Tesque), 2.5 mM EGTA- KOH (Sigma), 1 mM phenylmethylsulfonyl fluoride (PMSF, Nacalai Tesque)]. The whole cell lysates were subjected to SDS–polyacrylamide gel electrophoresis (PAGE) and electrotransferred onto polyvinylidene difluoride membranes (PVDF; Pall Corporation, Port Washington, NY, USA). Blocking to minimize non-specific interactions was done with Blocking One (Nacalai Tesque) or 5% BSA in Tween 20-containing Tris-buffered saline (TTBS) [20 mM Tris–HCl (pH 7.5), 137 mM NaCl, and 0.1% Tween 20] at room temperature for 30 min. Later, the membranes were incubated with the antibodies, which were diluted with TTBS containing 5% Blocking One or 3% BSA. Clarity (Bio-Rad) was used as the chemiluminescence substrate. A ChemiDoc XRSplus image analyzer (Bio-Rad) was used for the chemiluminescence detection and band intensity analysis.

### Immunoprecipitation

Immunoprecipitation of SH2D4A from mitotic cells were performed. The cells were arrested at mitosis by treatment with 5 µM S-Trityl-L-cysteine (STLC) (164,739; MilliporeSigma), which inhibits Eg5 kinesin motor protein, for 16 h. After mitotic shake-off, the collected mitotic cells were solubilized at 4 °C for 10 min in 1% Triton lysis buffer (25 mM HEPES–NaOH, 2 mM EDTA-NaOH, 5% glycerol, 50 mM NaF, 2 mg/ml aprotinin, 0.8 mg/ml pepstatin A, 2 mg/ml leupeptin, 2 mM PMSF, 20 mM β-glycerophosphate, 10 mM Na_3_VO_4_, 1% Triton X-100), followed by centrifugation to remove insoluble materials. The resulting lysates were subjected to immunoprecipitation using protein G-Sepharose beads (GE Healthcare, Waukesha, WI, USA) pre-coated with mouse IgG (sc-2025; Santa Cruz Biotechnology) or anti-SH2D4A (sc-514170; Santa Cruz Biotechnology) antibodies. 3-h after the incubation at 4 °C, the beads were washed three times with the Triton lysis buffer containing 0.01% Triton X-100. The immunoprecipitants were subjected to Western blotting analysis.

### Statistics

Statistical differences between the two datasets were analyzed using Student's *t*-test after analysis of variance by F-test. Statistical differences among more than two datasets were analyzed using one-way ANOVA with Tukey's post hoc test or Dunnett's post hoc test, or Welch's ANOVA with Games–Howell post hoc test, based on their variance that was analyzed using Bartlett's test. Statistical analysis was performed using the Microsoft Excel program (Microsoft, Redmond, WA, USA), EZR software (v.1.41; Saitama Medical Center, Jichi Medical University, Saitama, Japan)^55^, and R software (v.3.4.3; R Foundation for Statistical Computing, Vienna, Austria).

### Supplementary Information

Supplementary Information.

## Data Availability

The data used to support findings of the study are available from the corresponding author upon reasonable request.

## Abbreviations

3’UTR: 3’-Untranslated region
BSA: Bovine serum albumin
CDK1: Cyclin-dependent kinase 1
CIN: Chromosome instability
CRCs: Colorectal cancers
Dox: Doxycycline
FBS: Fetal bovine serum
γTuRC: γ-Tubulin ring complex
HCCs: Hepatocellular carcinomas
IB: Immunoblotting
IF: Immunofluorescence
MSS: Micro satellite stable
MYPT1: Myosin phosphatase targeting subunit 1
Nek2: NIMA-related kinase 2
PAGE: SDS–polyacrylamide gel electrophoresis
PCM: Pericentriolar material
PLK1: Polo-like kinase 1
PMSF: Phenylmethylsulfonyl fluoride
PP1: Protein phosphatase 1
PP2A: Protein phosphatase 2A
PVDF: Polyvinylidene difluoride membranes
SAC: Spindle assembly checkpoint
SDS: Sodium dodecyl sulfate
Ser/Thr: Serine/Threonine
SH2D4A: SH2 domain-containing protein 4A
siRNA: Small interfering RNA
STLC: S-Trityl-L-cysteine
TTBS: Tween 20-containing Tris-buffered saline
