# Publisher Correction: SH2D4A promotes centrosome maturation to support spindle microtubule formation and mitotic progression

**DOI:** 10.1038/s41598-024-53925-0

**Published:** 2024-02-29

**Authors:** Ryuzaburo Yuki, Yuki Ikeda, Ryuji Yasutake, Youhei Saito, Yuji Nakayama

**Affiliations:** Department of Biochemistry & Molecular Biology, Kyoto Pharmaceutical University, 5 Misasagi-Nakauchi-Cho, Yamashina-Ku, Kyoto, 607-8414 Japan

Correction to: *Scientific Reports* 10.1038/s41598-023-29362-w, published online 04 February 2023

The original version of this Article contained an error in the right panel of Figure 1F where labels of used antibodies were omitted. The omitted labels were respectively; anti-SH2D4A, anti-HA, and anti-α-tubulin. The original Figure 1 and accompanying legend appear below.

**Figure 1:**
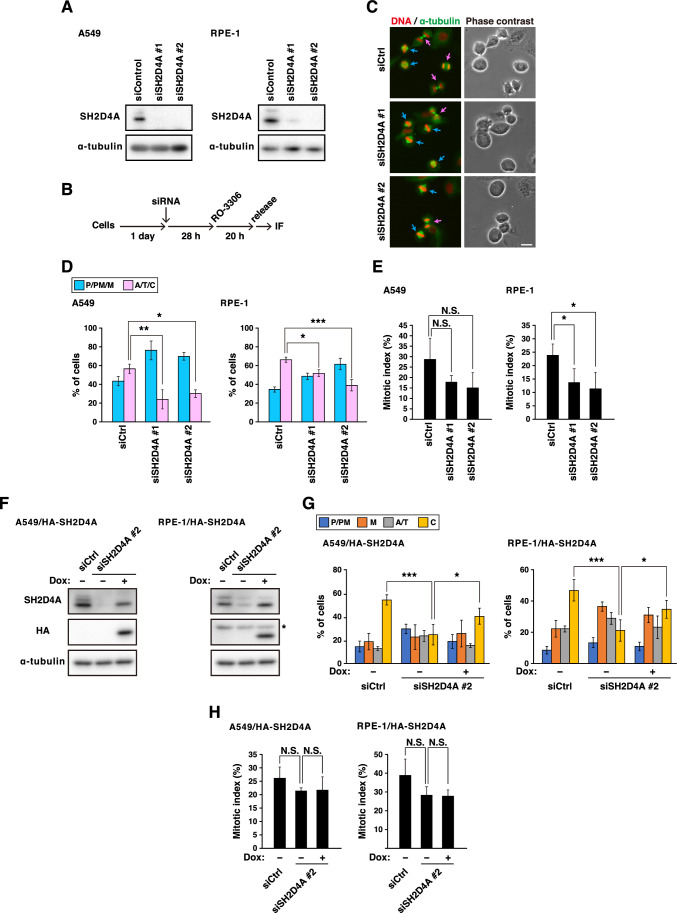
SH2D4A knockdown delays mitotic progression. (**A**) A549 or RPE-1 cells were transfected with control siRNA (siCtrl) or SH2D4A-targeting siRNAs (siSH2D4A #1 and #2). At 48 h after transfection, Western blot analysis was performed with indicated antibodies. (**B**–**E**) At 28 h after siRNA transfection, A549 or RPE-1 cells were treated with 6 or 8 µM RO-3306 for 20 h, washed with PBS(+), and cultured for 1 h or 45 min, respectively. Then, the cells were fixed and stained for α-tubulin (green) and DNA (red). (**B**) A schematic depiction of the synchronization method is shown. (**C**) Representative images of mitotic A549 cells are shown. The mitotic cells were classified into two groups (see “Methods”). Blue and pink arrows show the cells before and after anaphase onset, respectively. Scale bar, 20 µm. The percentages of cells of each group (**D**) or the mitotic indices (**E**) are plotted as the mean ± SD of more than three independent experiments (n > 168 in panel (**D**), n > 999 in panel (**E**)). (**F**) A549 or RPE-1 cells expressing inducible HA-SH2D4A (A549/HA-SH2D4A or RPE-1/HA-SH2D4A) were transfected with siCtrl or siSH2D4A #2 with or without 0.5 µg/mL (A549/HA-SH2D4A) or 0.3 µg/mL (RPE-1/HA-SH2D4A) Doxycycline (Dox). At 48 h after transfection, Western blot analysis was performed as in panel A. An asterisk indicates a non-specific band. (**G**, **H**) At 28 h after siRNA transfection, A549/HA-SH2D4A or RPE-1/HA-SH2D4A cells were treated with 6 or 8 µM RO-3306 for 20 h with or without Dox, washed, and cultured for 1 h or 50 min, respectively. The cells were stained for α-tubulin and DNA, and the mitotic cells were classified into four groups (see “Methods”) The percentages of cells of each group (**G**) or the mitotic indices (**H**) are plotted as the mean ± SD of four independent experiments (n > 214 in panel G, n > 1000 in panel (**H**)). Asterisks indicate significant differences (Dunnett’s test in panel (**D**) and (**E**), Tukey’s test in panel (**G**) and (**H**), * *p* < 0.05; ** *p* < 0.01; *** *p* < 0.001; N.S., not significant). Full blots are shown in Fig. S4.

Additionally, in Figure 5A, the labels of used antibodies were incorrect; the labels now read respectively; anti-SH2D4A, anti-PP1α, anti-PP1β, and anti-PP1γ. The original Figure 5A and accompanying legend appear below.

**Figure 5:**
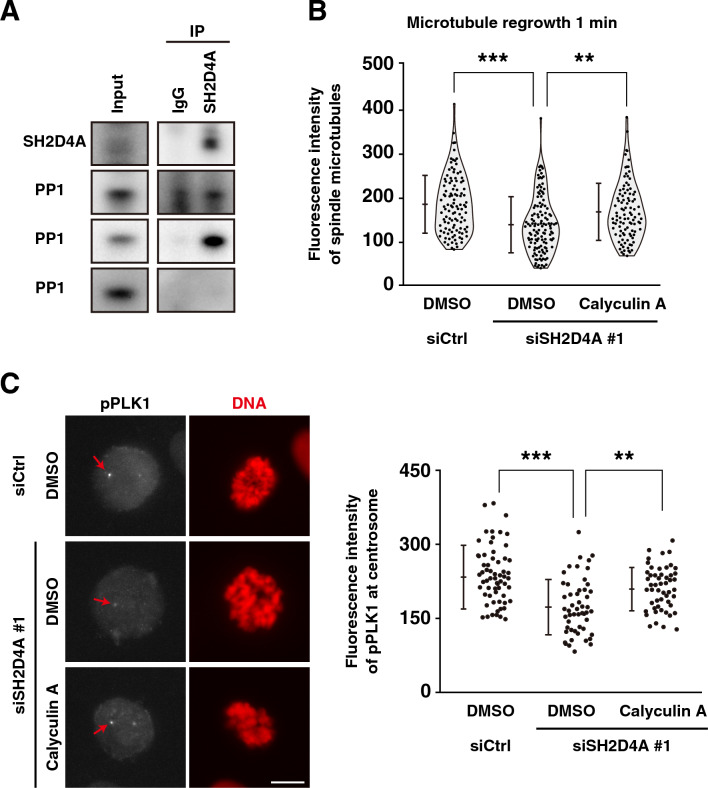
SH2D4A-mediated regulation of active PLK1 accumulation at centrosomes and microtubule nucleation through binding to PP1. (**A**) A549 cells were treated with 5 µM STLC for 16 h to arrest at mitosis. SH2D4A immunoprecipitates were subjected to Western blot analysis using indicated antibodies. (**B**, **C**) A549 cells were transfected with siCtrl or siSH2D4A #1. (**B**) At 28 h after siRNA transfection, cells were treated with 6 µM RO-3306 for 20 h, washed, cultured in fresh media for 10 min, and incubated on ice for 4 h with or without 2 nM calyculin A during the last 30 min. After incubation at 37 °C for 1 min with or without calyculin A, the cells were fixed and stained for α-tubulin and DNA. The fluorescence intensity of α-tubulin was measured and plotted as the mean ± SD from a representative experiment of two independent experiments (n > 100). Violin plots with data points are also shown. (**C**) At 28 h after siRNA transfection, cells were treated with 6 µM RO-3306 for 20 h with or without 2 nM calyculin A during the last 30 min. RO-3306-treated cells were washed, cultured in fresh media for 30 min with or without calyculin A. Then, the cells were fixed and stained for phospho-PLK1 (gray) and DNA (red). (Left) Representative images of prometaphase cells are shown. The focus was set to one of the centrosomes, and the red arrows indicate the centrosome. Scale bar, 10 µm. (Right) The fluorescence intensity of phospho-PLK1 at centrosomes in prometaphase per cell was measured and the average between the two centrosomes was plotted as the mean ± SD from a representative experiment of two independent experiments (n > 52). Asterisks indicate significant differences. Asterisks indicate significant differences (Tukey’s test, ** *p* < 0.01; *** *p* < 0.001). Full blots are shown in Fig. S4.

The original Article has been corrected.

