# Supplementary material for: SH2D4A promotes centrosome maturation to support spindle microtubule formation and mitotic progression
Source: Sci Rep. 2023 Feb 4;13:2067. doi: 10.1038/s41598-023-29362-w (PMC9899277; doi:10.1038/s41598-023-29362-w)
Supplement: Supplementary file 1 — Supplementary Information. [file 41598_2023_29362_MOESM1_ESM.pdf]

Supporting information

Fig. S1. The effect of SH2D4A knockdown on mitotic entry after release from RO-3306 block.

At 28 hours after siRNA transfection, A549 or RPE-1 cells were treated with 6 or 8 μM RO-3306 for 20 hours, washed with PBS(+), and cultured in fresh media for 5 or 10 minutes, respectively. Then, the cells were fixed and stained for α-tubulin and DNA. (**A**) The number of prophase, prometaphase, and metaphase cells of A549 cells was counted, and the percentages of cells of each group are plotted as the mean ± SD of three independent experiments (n > 201). (**B, C**) The mitotic indices of A549 (B) or RPE-1 (C) cells, are plotted as the mean ± SD of three independent experiments, respectively (n > 999 in each experiment). Asterisks indicate significant differences (Dunnett's test, * $p < 0.05$, ** $p < 0.01$; N.S., not significant).

Fig. S2. Localization of HA-tagged SH2D4A in mitosis.

(**A**) A549 cells were treated with 5 μM STLC for 16 hours to arrest at mitosis, and whole cell lysates were obtained and analyzed by Western blot with indicated antibodies. (**B**) A549/HA-SH2D4A cells were treated with or without 5 μg/mL Dox for 20 hours, and whole cell lysates were obtained and analyzed by Western blot with indicated antibodies. An asterisk indicates a non-specific band. Full blots are shown in Fig. S4. (**C**) A549/HA-SH2D4A cells were treated with 6 μM RO-3306 for 20 hours with or without Dox, washed with PBS(+), and cultured in fresh media for 30 minutes. Then, the cells were fixed and stained for HA-SH2D4A, α-tubulin, and DNA. Scale bar, 10 μm.

Fig. S3. Confirmation of calyculin A treatment.

A549 cells were treated with or without 2 nM calyculin A for 30 minutes. Whole cell lysates were obtained and analyzed by Western blot with anti-pSer/Thr and anti-α-tubulin antibodies. Full blots

are shown in Fig. S4.

Fig. S4. Full-length blots.

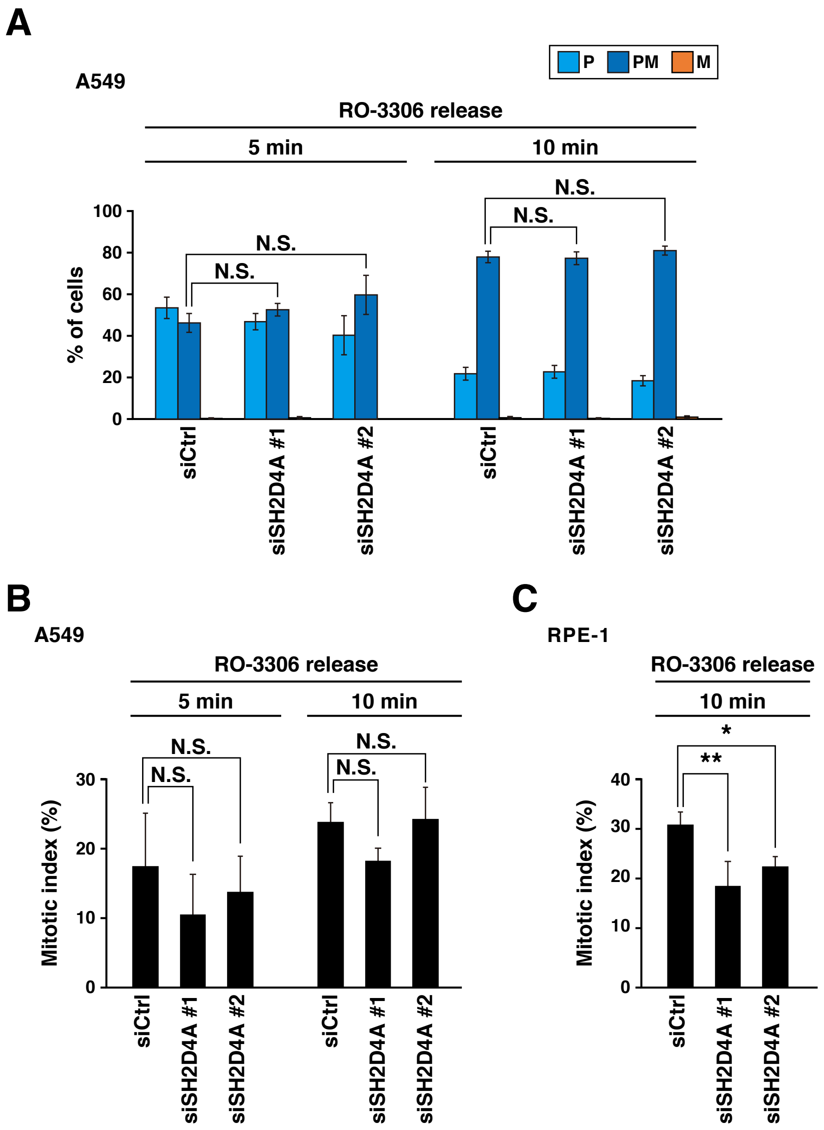

Supplementary figure 1

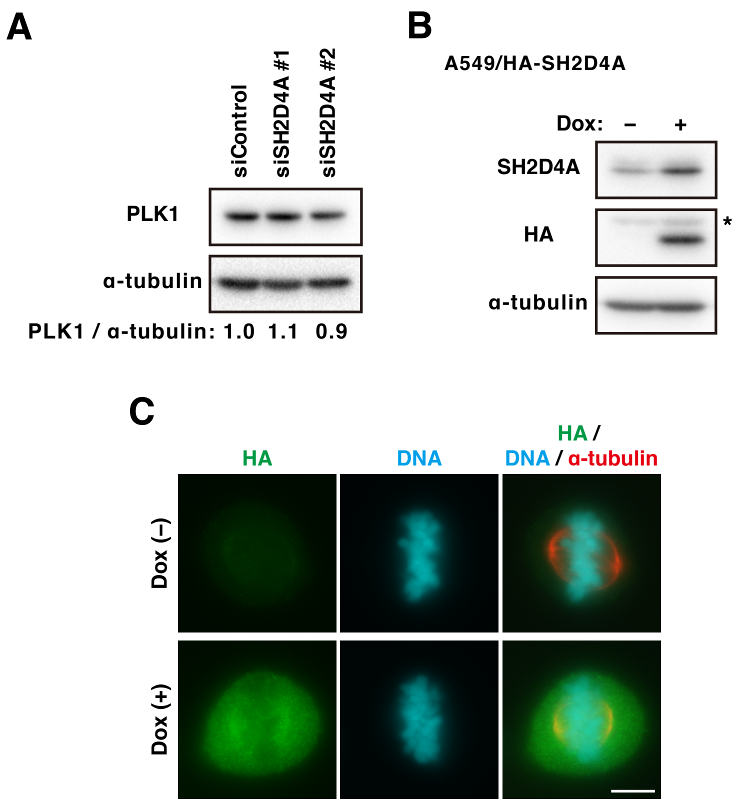

Supplementary figure 2

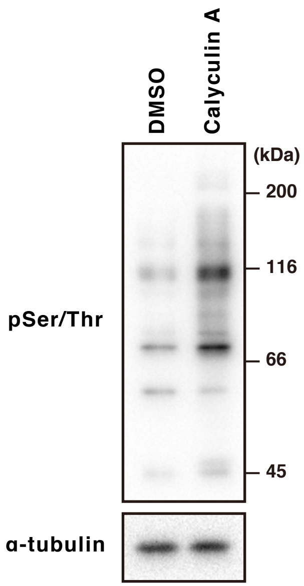

Supplementary figure 3

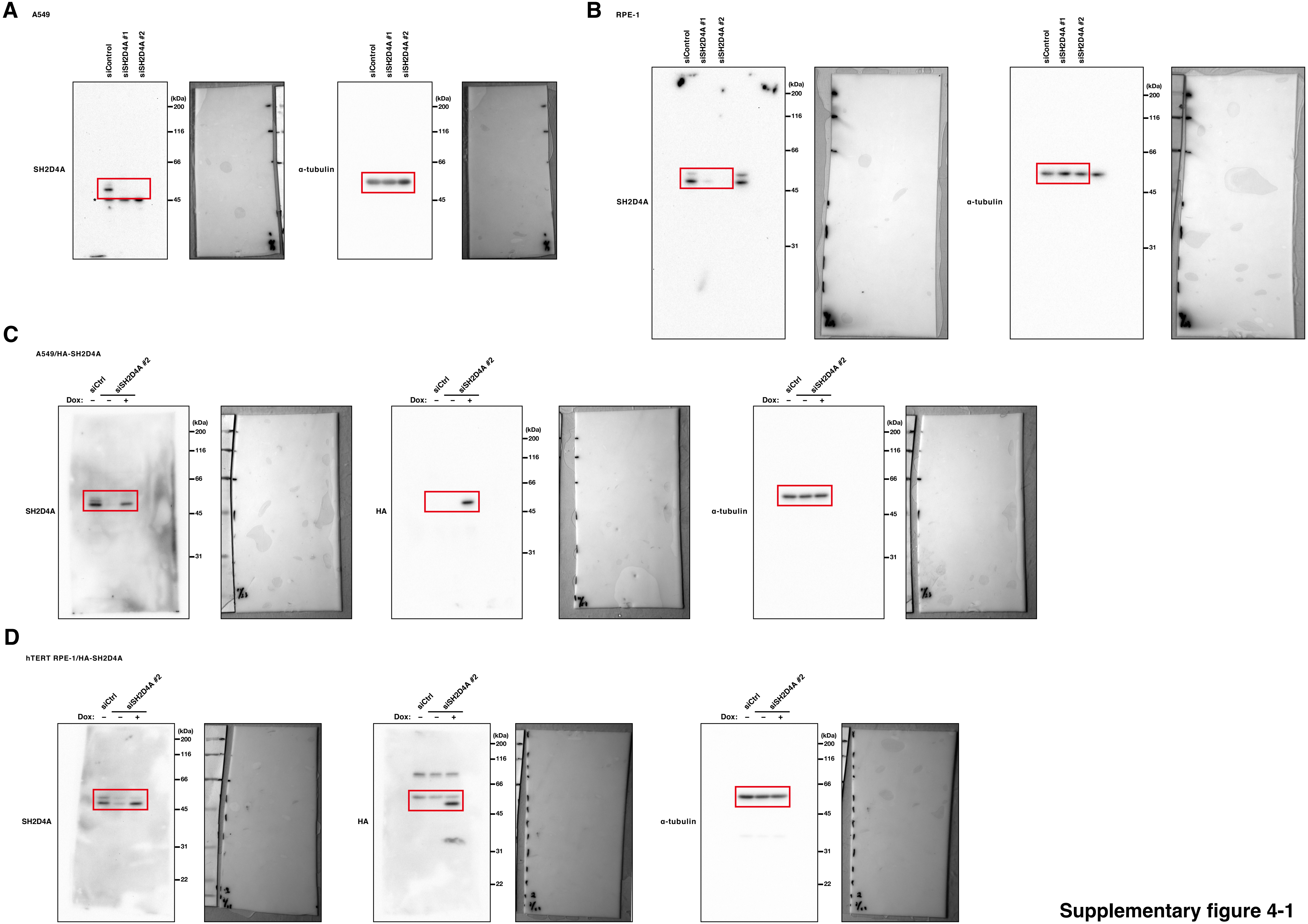

Supplementary figure 4-1

E

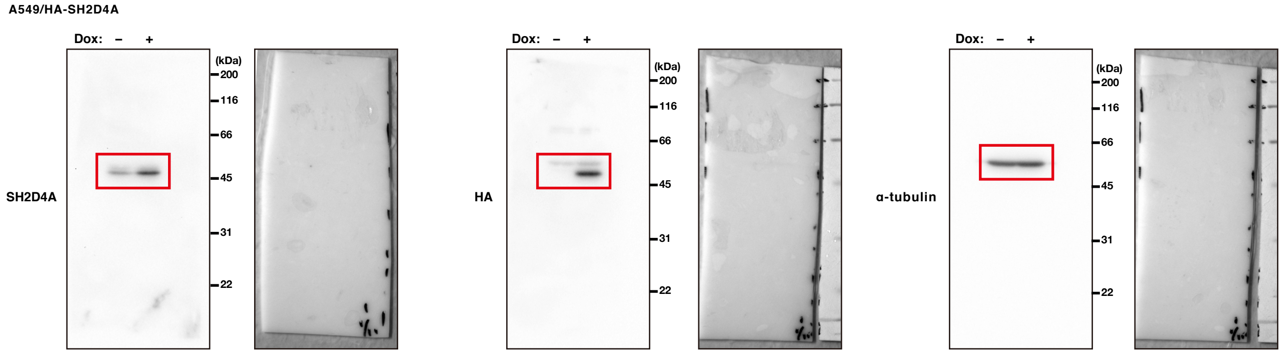

F

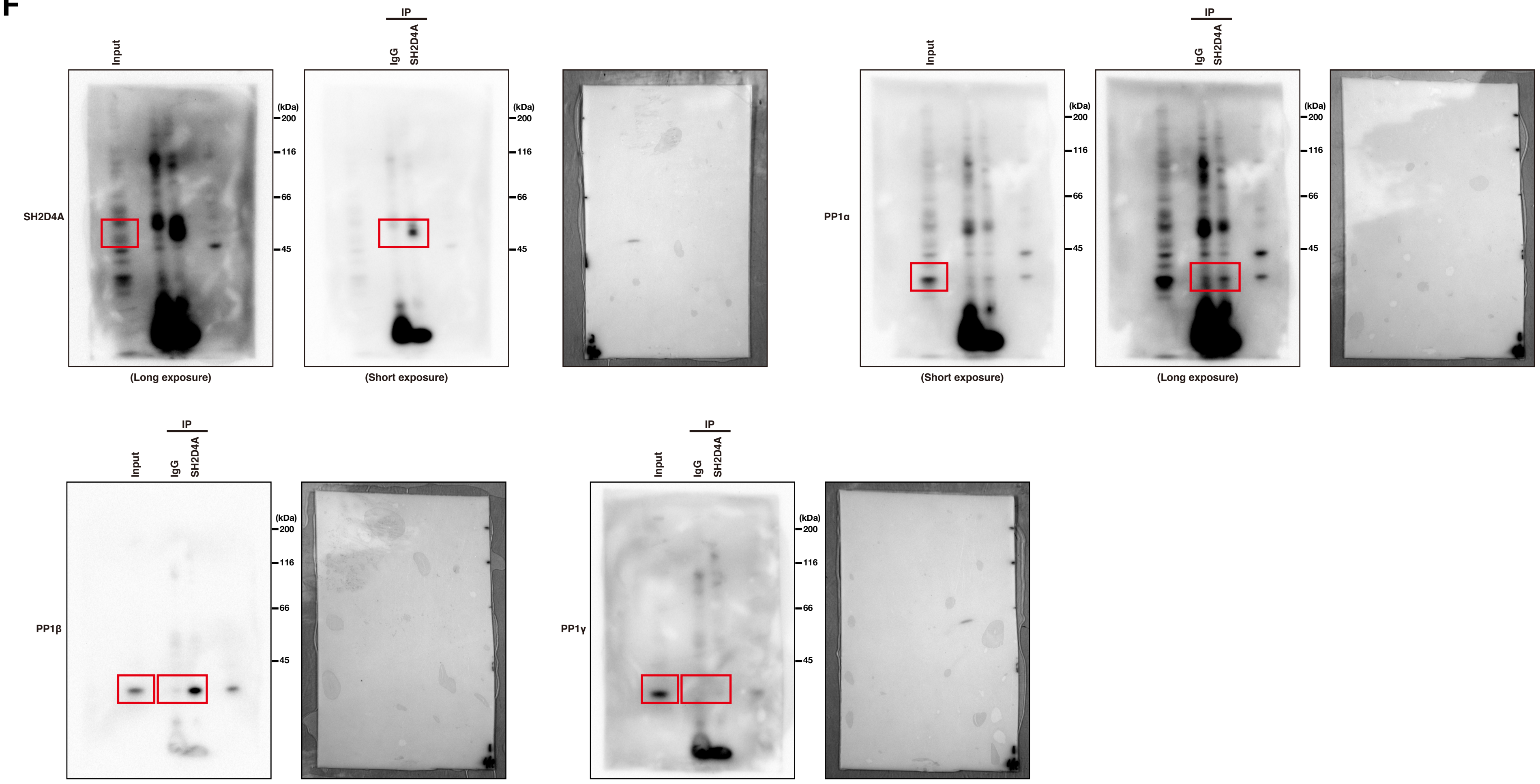

Supplementary figure 4-2

G

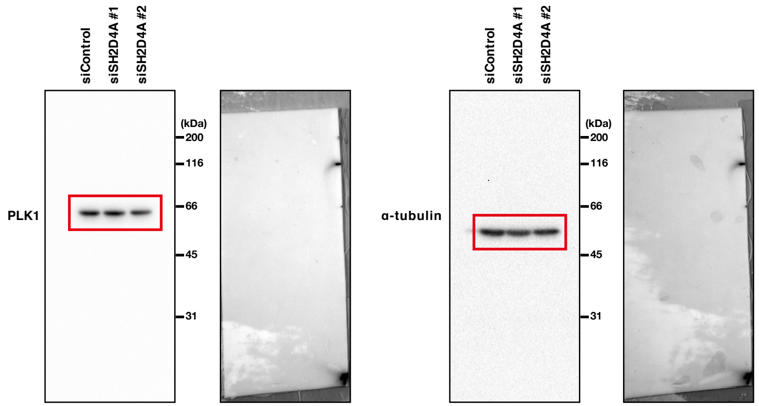

H

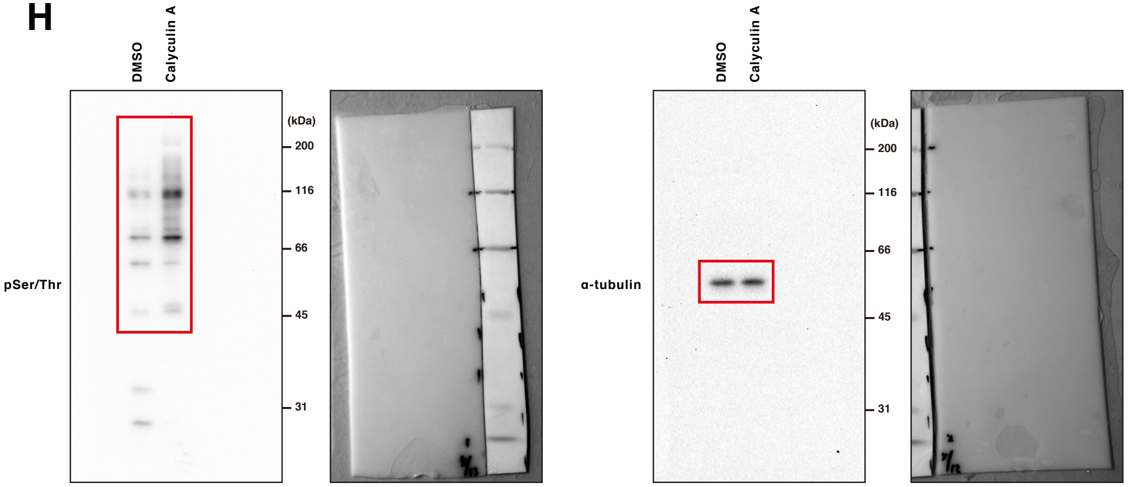

Supplementary figure 4-3
